# Fucoidan Purified from *Sargassum polycystum* Induces Apoptosis through Mitochondria-Mediated Pathway in HL-60 and MCF-7 Cells

**DOI:** 10.3390/md18040196

**Published:** 2020-04-08

**Authors:** Ilekuttige Priyan Shanura Fernando, Kalu Kapuge Asanka Sanjeewa, Hyo Geun Lee, Hyun-Soo Kim, Andaravaas Patabadige Jude Prasanna Vaas, Hondamuni Ireshika Chathurani De Silva, Chandrika Malkanthi Nanayakkara, Dampegamage Thusitha Udayangani Abeytunga, Dae-Sung Lee, Jung-Suck Lee, You-Jin Jeon

**Affiliations:** 1 Department of Marine Bio-Food Sciences, Chonnam National University, Yeosu 59626, Korea; shanurabru@gmail.com; 2 Department of Marine Life Science, Jeju National University, Jeju 690-756, Korea; asanka.sanjeewa001@gmail.com (K.K.A.S.); hyogeunlee92@gmail.com (H.G.L.); 3 National Marine Biodiversity Institute of Korea, 75, Jangsan-ro 101-gil, Janghang-eup, Seocheon 33662, Korea; gustn783@mabik.re.kr; 4 Department of Chemistry, University of Colombo, Colombo 3, Colombo 00700, Sri Lanka; prasannarokx@gmail.com (A.P.J.P.V.); hicdesilva@gmail.com (H.I.C.D.S.); abeytunga@gmail.com (D.T.U.A.); 5 School of Natural Sciences, University of Tasmania, Private Bag 75, Hobart, Tasmania 7001, Australia; 6 Department of Plant Sciences, University of Colombo, Colombo 3, Colombo 00700, Sri Lanka; hewa_nana@yahoo.com; 7 Department of Applied Research, National Marine Biodiversity Institute of Korea, 75, Jangsan-ro 101-gil, Janghang-eup, Seocheon 33662, Korea; daesung@mabik.re.kr; 8 Research Center for Industrial Development of Seafood, Gyeongsang National University, Tongyeong 53064, Korea; 9 Marine Science Institute, Jeju National University, Jeju Self-Governing Province 63333, Korea

**Keywords:** Sri Lankan algae, anticancer, sulfated polysaccharide, fucoidan, Celluclast, sargassum

## Abstract

Fucoidans are biocompatible, heterogeneous, and fucose rich sulfated polysaccharides biosynthesized in brown algae, which are renowned for their broad-spectrum biofunctional properties. As a continuation of our preliminary screening studies, the present work was undertaken to extract polysaccharides from the edible brown algae *Sargassum polycystum* by a modified enzyme assisted extraction process using Celluclast, a food-grade cellulase, and to purify fucoidan by DEAE-cellulose anion exchange chromatography. The apoptotic and antiproliferative properties of the purified fucoidan (F5) were evaluated on HL-60 and MCF-7 cells. Structural features were characterized by FTIR and NMR analysis. F5 indicated profound antiproliferative effects on HL-60 leukemia and MCF-7 breast cancer cells with IC_50_ values of 84.63 ± 0.08 µg mL^−1^ and 93.62 ± 3.53 µg mL^−1^ respectively. Further, F5 treatment increased the apoptotic body formation, DNA damage, and accumulation of HL-60 and MCF-7 cells in the Sub-G_1_ phase of the cell cycle. The effects were found to proceed via the mitochondria-mediated apoptosis pathway. The Celluclast assisted extraction is a cost-efficient method of yielding fucoidan. With further studies in place, purified fucoidan of *S. polycystum* could be applied as functional ingredients in food and pharmaceuticals.

## 1. Introduction

Fucoidans found in brown algae are documented for their broad spectrum of bioactive properties, including anticancer, anticoagulant, antioxidant, anti-inflammatory, and immunomodulatory activities beneficial for the pharmaceutical, cosmeceutical, nutraceutical, and functional food industries [1]. The structure of fucoidan comprises of alternating units of L-fucose with mannose, glucose, galactose, and xylose monosaccharides with substituted sulfate groups. The primary linkage between α-l-fucopyranosyl residues is the (1→3) and (1→4) with sulfate groups substituted at either C2, C4, or both positions [1,2]. However, differences in fucoidan structure have been observed in different brown algae and remain ambiguous for their structures are highly heterogeneous. Typically, the structural characterization of fucoidan is accomplished by analyzing the monosaccharides composition, their connectivity, and substituted functional groups (methylation analysis, FTIR, and NMR) [3]. The publication by Bilan et al. (2013) reveals some significant findings regarding the fucoidan structure of *S. polycystum* harvested from Vietnam, which is comprised of 3-linked α-l-fucopyranose 4-sulfate residues [4]. Apart from the species specificity, fucoidan structure could vary depending on environmental factors and stress conditions. The structure of fucoidan (molecular weight, monosaccharide composition, and their sequence, degree, and substitution of sulfate groups) is a critical factor that determines its biofunctional properties. Li et al. (2008) provide a detailed review of up to date understanding regarding the structure-activity relationship of fucoidans [5]. By far, the anticancer properties of fucoidan have been found to increase with the increasing degree of sulfation and with reduction of molecular weight up to a certain limit. 

Cancer has become a burden and a leading cause of death throughout the world. The number of cancer incidents in 2012 reached approximately 14.1 million, among which 8.2 million deaths have been reported [6]. The rapid growth of cancer incidents is predicted to be due to the increasing population and modernizing lifestyle patterns. Consumption of foods with artificial coloring, flavoring, and preservatives, use of tobacco, physical inactivity, exposure to ionizing radiation, and carcinogenic substances are among the major factors that contribute to the development of cancer [7]. Though a vast number of anticancer drugs are available, continuing the search for new cancer medicine is a prevailing task as many available anticancer agents cause serious side effects on normal tissues. The lack of selectivity is one of the major drawbacks in many of these drugs except for a limited number of anticancer medications such as “vemurafenib” that show their effects on melanoma cells with V600E BRAF mutation and monoclonal antibodies that specifically target cancer antigens [8]. The heterogeneous nature of cancers and multidrug resistance causes the development of anticancer drugs a challenging task [9]. Fucoidans, by far, have been recognized as a biocompatible substance that indicates positive biological responses. A recent study by Silchenko et al. (2017) describes the anticancer activity of fucoidan and sulfated fucooligosaccharides from *Sargassum horneri,* whereas the fucoidans are reported to suppress the colony formation of DLD-1 (colorectal adenocarcinoma) cells [10]. Fucoidans mediate anticancer functions not only by regulating intracellular mechanisms of cancer cells such as apoptosis, but also the activity of immune cells such as lymphocytes and natural killer cells [8,11]. Apart from the direct chemotherapeutic effects, fucoidan rich polysaccharides have shown radioprotective effects that make them a useful substance in cancer-radiotherapy [12]. The objective of the current study was to explore the anticancer activity of fucoidan rich polysaccharides of untapped *S. polycystum* harvested from the tropical island, Sri Lanka, and to explore its structural properties. Recently Palanisamy et al. (2017) have reported the anticancer potential of a fucoidan extract from *S. polycystum* on MCF-7 cells [13]. The present study so far is the first to report modified enzyme assisted extraction of fucoidan enriched polysaccharides from *S. polycystum*, anion exchange chromatographic purification, and detailed information of their anticancer properties.

## 2. Results

### 2.1. Yields and Chemical Composition of the Polysaccharide Extracts and Fractions

Based on the compositional analysis (AOAC methods) *S. polycystum* indicated 2.75 ± 0.08% moisture, 17.62 ± 0.16% ash, 52.46 ± 0.52% carbohydrate, 17.36 ± 0.04% protein, and 1.85 ± 0.06% lipids on a dry basis. The yield of ethanol precipitate was 6.88 ± 0.52%. The ash content of the ethanol precipitate was 0.08 ± 0.01% (relative to the dry weight of raw material).

### 2.2. Purification of Polysaccharides Molecular Weight Distribution and Monosaccharide Composition

**Figure 1:**
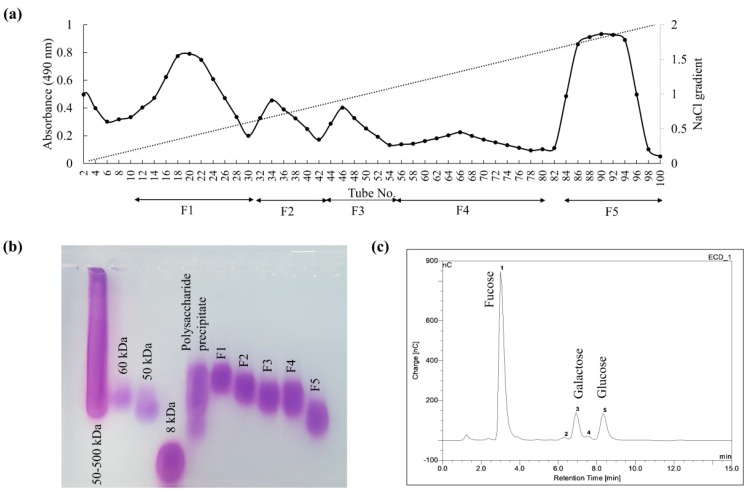
Purification of precipitated polysaccharides by DEAE-cellulose anion exchange chromatography. (**a**) Separating the collected column eluates into five fractions based on their polysaccharide content, (**b**) molecular weight distribution of the eluted column fractions (F1–F5), and (**c**) the monosaccharide composition analysis of the fraction F5.

As indicated in Figure 1a, anion exchange purification resolved the polysaccharide precipitate into five fractions (F1–F5). The recovery yield of F1 and F5 was higher than other fractions. The molecular weight distribution of fractions (F1–F5) indicated a decreasing trend and was estimated to be centered on 77.0, 65.5, 59.5, 60.0, and 39.5 kDa, respectively (Figure 1b). The monosaccharide composition of each fraction is given in Table 1. F1 had a higher galactose and mannose content, whereas the other successive fractions indicated increasing amounts of fucose. Fucose content was highest in F5 (Figure 1c) with galactose and a minor amount of glucose.

### 2.3. Characterization of Polysaccharide Structure (FTIR and NMR Analysis)

**Figure 2:**
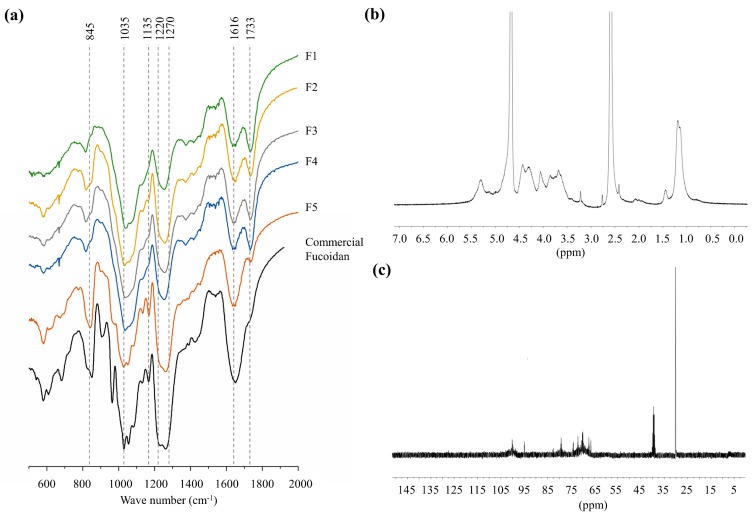
Structural characterization of polysaccharide fractions. (**a**) FTIR spectra of the fractions (F1–F5) provided in comparison to the commercial fucoidan, (**b**) ^1^H NMR spectrum of F5, and (**c**) ^13^C NMR spectrum of F5. The NMR spectra were obtained for the deuterium exchanged polysaccharides.

Wavenumber range 400–2000 cm^−1^ of FTIR spectra covering the fingerprint region of polysaccharides were used to characterize the structural properties of polysaccharides. All FTIR spectra, including column fractions and the commercial fucoidan standard, indicated prominent peaks at 845, 1035, 1616 cm^−1,^ and a broad peak between 1220–1270 cm^−1^. A clear difference was seen for the intensity of 1220–1270 cm^−1^ peak between each spectrum, although the intensity of the peak at 1035 cm^−1^ was similar between all. The peak at 1733 cm^−1^ was absent in the commercial fucoidan, whereas a minor peak was recorded for F5. However, 1733 cm^−1^ peak was prominent in all other fractions (F1–F4). ^1^H and ^13^C NMR spectra of deuterium exchanged F5 fraction are presented in Figure 2b,C. In the ^1^H spectrum, solvent peaks were observed at 2.50 ppm for dimethyl sulfoxide and 4.80 ppm for deuterium oxide. Unresolved peaks between 5.1–5.4 ppm could be assigned to protons of α-l-fucopyranosyl units. Prominent peaks observed at 4.46, 3.90, and 3.71 ppm were respectively assigned to H-1, H-5, and H-3 of D-galactons. The two overlapping peaks at 1.15, and 1.4 ppm were assigned to methyl groups in fucose [1,2]. In the ^13^C spectrum, prominent peak 101.6 and peaks between 65–80 are arising from the (1–6)-β-d-linked galactons [2].

### 2.4. Variation of Antiproliferative Activity of the Column Fractions

**Figure 3:**
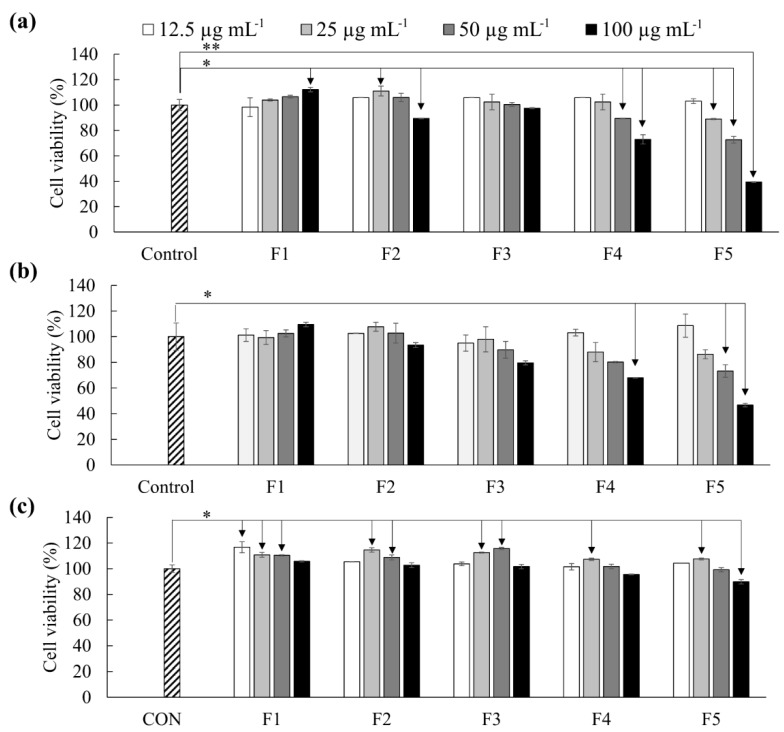
Antiproliferative activity of polysaccharide fractions as a measure of cell viability. (**a**) HL-60, (**b**) MCF-7, and (**c**) Vero cells. Cells were pre-seeded in 96 well plates for 24 h and incubated with samples for another 24 h. Cell viability was measured by MTT assay. Results are given as the means ± SD (n = 3). Significant differences from the control were identified at * *p* < 0.05 and ** *p* < 0.001.

As shown in Figure 3, F4 and F5 promptly reduced the viability of both HL-60 and MCF-7 cells compared to other fractions. The antiproliferative effects of F5 were stronger than F4. The IC_50_ values related to F5 treatment was 84.63 ± 0.08 µg mL^−1^ and 93.62 ± 3.53 µg mL^−1^ respectively on HL-60 and MCF-7 cells. The viability of Vero cells (Figure 3c) was simultaneously evaluated to compare the cytotoxic effects of these polysaccharides on non-cancerous cells. The viability of Vero cells was 89.83 ± 1.76% at 100 µg mL^−1^ concentration.

### 2.5. Effect of F5 upon Apoptotic Body Formation in HL-60 and MCF-7 Cells and Pathway Studies by Western Blot Analysis

**Figure 4:**
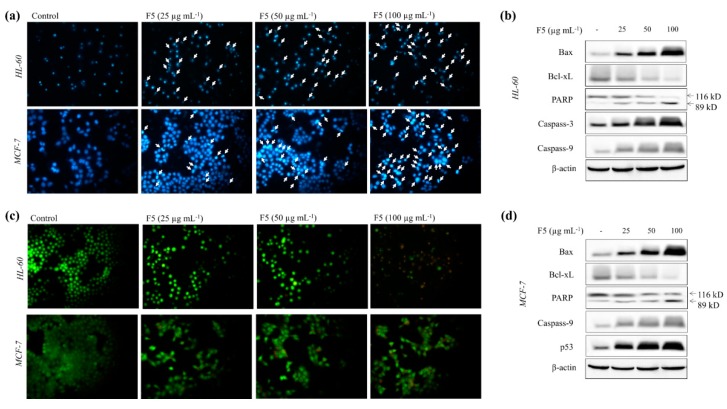
Effects of F5 in inducing apoptotic body formation in HL-60 and MCF-7 cells and analysis of the levels of molecular mediators. (**a**) Cells under Hoechst 33342 staining, (**b**) Western blot analysis of the levels of apoptosis-related molecular mediators in HL-60 cells, (**c**) cells under nuclear double staining, and (**d**) Western blot analysis of the levels of apoptosis-related molecular mediators in MCF-7 cells. Experiments were repeated three times to confirm the reproducibility.

As shown in Figure 4a, under the Hoechst 33342 staining, HL-60 and MCF-7 cells indicated increased nuclear fragmentation and condensation (intensified spots) for increasing F5 concentrations. Nuclear double staining method (Figure 4c) assists in distinguishing early and late apoptosis or necrosis. Green fragmented nuclei (early apoptosis) were seen under 25 µg mL^−1^ of F5 treatment after a 24 h incubation period, which increased with increasing F5 concentrations. The presence of orange color spots together with green color fragmented nuclei (late apoptosis) were detected under 50 µg mL^−1^ concentration. Increased late apoptosis events were observed with 100 µg mL^−1^ of F5 concentration, whereas a few necrotic HL-60 cells were also detected. Western blot results indicated increased production of Bax, caspases, and p53 (only MCF-7) levels with increasing F5 concentrations together with increased PARP cleavage. Alternatively, reducing levels of Bcl-xL were also detected with increasing F5 concentrations.

### 2.6. F5 Increased the DNA Damage in HL-60 and MCF-7 Cells and the Population of Sub-G_1_ Hyperploid Cells

**Figure 5:**
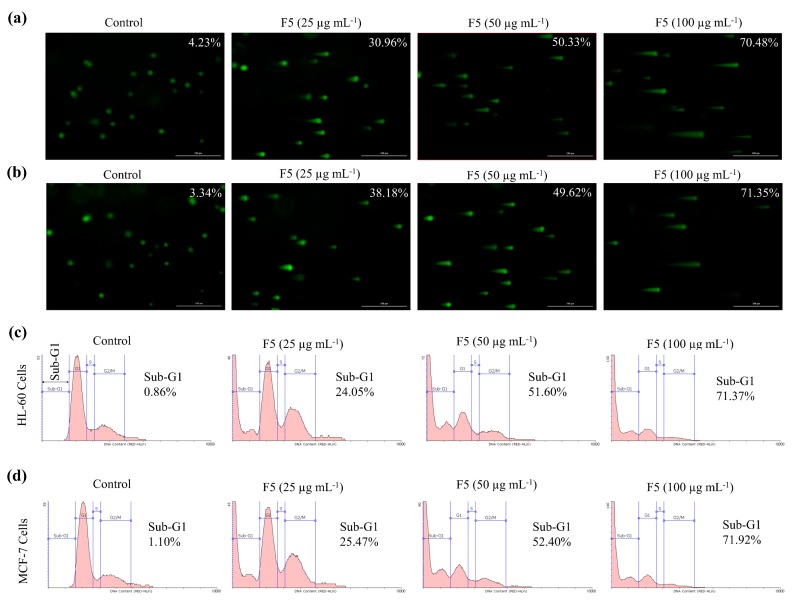
Effects of F5 in inducing single-cell DNA damage in HL-60 and MCF-7 cells and cell cycle analysis. Comet assay (**a**) HL-60 cells, (**b**) MCF-7 cells. Cell cycle analysis of (**c**) HL-60 cells, and (**d**) MCF-7 cells. Pre-seeded cells were exposed to different concentrations of F5 for 24 h. Experiments were repeated three times to confirm the reproducibility.

Comet assay serves as a tool in identifying cellular DNA damage. As depicted in Figure 5a,b, the length of the comet tail increased in both HL-60 and MCF-7 cells with increasing F5 concentrations. Flow cytometric analysis with propidium iodide (PI) is widely employed in identifying the accumulation of cells in different phases of cell cycle based on their DNA content [14]. According to Figure 5c,d, the proportion of Sub-G_1_ apoptotic cells increased with increasing F5 concentrations.

## 3. Discussion

Cancer is one of the leading causes of death throughout the world, which is predicted to increase in the future. The need for anticancer agents is an ever-increasing necessity, as many anticancer drugs cause serious side effects related to their inherent toxic effects. The literature suggests that fucoidan from brown algae could be a potential anticancer agent with promising bioactive effects and biocompatible properties [15]. A recent clinical study suggests that the administration of low molecular weight fucoidans to patients undergoing chemotherapy for metastatic colorectal cancer significantly improved the disease control rate compared to the control group [16]. The bioactive properties of fucoidan largely depend upon its structural characteristics. The structure of fucoidan is highly heterogeneous and indicates variation based on its source. In a previous study, fucoidan obtained from *S. polycystum* by hot water extraction has shown moderate antioxidant activity for DPPH scavenging, total antioxidant and reducing power assays, and anticancer activity in MCF-7 cells with an IC_50_ of 50 μg mL^−1^ [13]. Current investigations focus on the evaluation of antiproliferative properties of polysaccharides, and a purified fucoidan fraction from the Celluclast assisted extraction of *S. polycystum*. The selection of enzyme-assisted extraction supposedly gives better extraction yields compared to the conventional water extraction of polysaccharides. Here Celluclast, a food-grade cellulase, assists in hydrolyzing cellulose in cell walls [17]. Hexane washing removes the lipids and nonpolar pigments, whereas ethanol assists in removing un-bound phenolic compounds. Formaldehyde pretreatment causes polymerization of phenolic compounds that remain bound to the polysaccharide matrix and with other polar molecules via strong intermolecular interactions [1]. This prevents the water-based extract from getting contaminated by phenolic compounds. pH value is another factor that determines the solubility of anionic and cationic polysaccharides. Brown algae are rich in anionic polyelectrolyte alginic acid (pKa around 3.4–4.4) unlike other than fucoidan (pKa around 1.0–2.5) [18]. The optimum extraction conditions of Celluclast (pH 4.5) hence minimize the solubility of high molecular weight alginic acid, thus preventing the contamination of intended fucoidan extraction. However, CaCl_2_ was incorporated into the extract at a later stage to facilitate the complexation and precipitation of any remaining alginic acid as calcium alginate [1]. Proteins are among the compounds soluble in water, which get precipitated with the addition of ethanol. To prevent the contamination of the final ethanol precipitate with proteins, we incorporated a commercial food grade protease (Alcalase) to the extraction mixture to facilitate the hydrolysis of proteins. Finally, ethanol was added to the neutralized and concentrated extraction mixture to lower its dielectric constant and precipitate polysaccharides. Essentially, the above extraction method, except for some minor modifications, was optimized during our previous studies to be efficient in purifying fucoidans [1]. The precipitated polysaccharides were subjected to anion exchange chromatography, where five fractions were obtained. Elution of the DEAE-Sepharose column with increasing Cl^-^ concentrations enabled the successive elution of less negative to highly negatively charged polysaccharides. The chemical composition of the fractions indicates increasing amounts of sulfate through the successive column fractions agreeable with the increasing negative charge of the polysaccharides. The total polyphenol and protein contents were negligible in the fractions. 

The molecular weight of sulfated polysaccharides is a major deterministic factor of its bioactivity [1]. The fractions indicated that different molecular weights ranged between 39–77 kDa. The lowest molecular weight was observed in F5, which indicated the best antiproliferative properties. Similar observations have reported that low molecular weight fucoidans are very effective as antiproliferative agents [5]. The composition of monosaccharides explicated substantial deviation from each other. An increase of the fucose content was observed with successive fractions, the highest being 71.96% in F5 with 12.31% of galactose (a galactofucan). L-fucose is the major monosaccharide constituent in fucoidans, which reports associating with sulfate groups. This agrees with the relatively high sulfate content in F5 compared to other fractions. Much evidence is, therefore, in agreement with F5 being a fucoidan. Crude fucoidan obtained from *S. polycystum* by Palanisamy et al. (2017) reported 46.8% fucose content with 22.35 ± 0.23% sulfate with minor amounts of other monosaccharides, galactose (14.3%), glucose (11.5%), and xylose (13.2%) [13]. Bilan et al. (2013) have described the purification (anion-exchange chromatography) and structural characterization of two highly sulfated galactofucan fractions from *S. polycystum* following desulfation, methylation analysis, Smith degradation, and partial acid hydrolysis following mass-spectrometric and NMR monitoring [4]. Their fine structure report consists of mainly 3-linked α-l-fucopyranose units with C4-sulfate substitution, which is common to fucoidans and short sequences of above structures interspersed by residues of single 2-linked α-d-galactopyranose with C4 sulfation which is rather an unusual configuration.

Vibrational spectroscopic data were used in the structural characterization of the polysaccharides as they provide information about functional groups. The prominent peaks at 845–1035 cm^−1^ and the broad peak between 1220–1270 cm^−1^ respectively, representing the bending vibration of C-O-S, stretching vibration of glycosidic (C-O-C) bridge (typical to all polysaccharides), and the stretching vibrations of S=O bond in sulfate groups [1]. Interestingly the broadened peak between 1220 and 1270 cm^−1^ indicated a higher intensity in both commercial fucoidan and F5 fraction, suggesting a higher sulfate substitution. The intensity of that peak increased with successive fractions agreeing with the increased sulfate content observed. The intense peak at 1616 cm^−1^ is resulting from the asymmetric carboxylate O-C-O vibration [19]. 

Based on MTT cell viability assay, F5 indicated cytotoxicity on HL-60 and MCF-7 cancer cells with IC_50_ values of 84.63 ± 0.08 µg mL^−1^ and 93.62 ± 3.53 µg mL^−1^ respectively. Nonmalignant monkey kidney epithelial cells (Vero) were the least sensitive. Previous studies report substantially different IC_50_ values for the anticancer activity of fucoidans. According to Isnansetyo et al. (2017), fucoidans purified (anion exchange chromatography) from three tropical algae *Sargassum cristaefolium*, *Turbinaria conoides,* and *Padina fraseri* have shown substantially different IC_50_ values for cytotoxicity on MCF-7 and WiDr cell lines [19]. Herein *P. fraseri* fucoidan has shown IC_50_ of 144 and 118 μg mL^−1^, respectively, on MCF-7 and WiDr cells, whereas the IC_50_ has ranged between 461 and 663 μg mL^−1^ for the fucoidans of *S. cristaefolium*, and *T. conoides*. 

There could be a number of possibilities for the observed antiproliferative effects such as apoptosis, necrosis, cell cycle arrest, or a combination of two or more of the above effects. Evaluation of nuclear morphology revealed that F5 treatment increased the apoptotic body formation with an increased number of cells in late apoptosis. Further, the accumulation of cells in the Sub-G1 phase of the cell cycle agrees with the observed formation of apoptotic bodies. As evident from comet assay, DNA fragmentation is a characteristic feature observed in cells undergoing apoptosis. Apoptosis is a cellular homeostatic process that eliminates damaged cells without causing damage to neighboring tissues [20]. Hence substances that could induce apoptosis in cancer cells receive greater attention in the discovery of anticancer drugs.

Apoptosis could be triggered by two main pathways, either the mitochondria-mediated intrinsic pathway or cell surface receptor-mediated extrinsic pathway. Sensitivity to a vast number of death stimuli makes mitochondria-mediated apoptosis the most frequently studied death mechanism. This mechanism is inactivated in many cancer cells. Thus, its activation ramifies the therapeutic perspectives of treating several malignant diseases [20]. The mitochondria-mediated apoptotic pathway is controlled via a complex web of signaling cascade. Some of the major signaling molecules involved are Bax, Bcl-xL, PARP, p53, and caspase-9 and -3. The pro-apoptotic protein, Bax and the anti-apoptotic protein, Bcl-xL, belong to Bcl-2 family proteins. The Bax/Bcl-xL ratio is considered a major prognostic factor of apoptosis. Activation of Bax causes disruptions in the voltage-dependent anion channels in mitochondria releasing pro-apoptotic factors and cytochrome c that propagate apoptosis. Bcl-xL, which resides on the outer mitochondrial membrane, inhibits the activation of pro-apoptotic proteins such as Bax, thereby inhibiting the release of pro-apoptotic factors and cytochrome c [21]. In the present study, treatment of F5 increased the levels of Bax while reducing Bcl-xL. Caspases are next in line where their activation is triggered by cytochrome c, initiator caspases such as caspase-8 and -9, or by autocatalytic processes. The activated effector caspases (caspase-3, -6, and -7) catalyze the cleavage of critical regulatory proteins, such as PARP, which is involved in maintaining genomic stability by regulating base-excision DNA repair. F5 markedly increased the levels of caspase-9 in both HL-60 and MCF-7 cells. Although caspase-3 is produced in HL-60 cells, MCF-7 cells cannot synthesize caspase-3 due to a deletion in the CASP-3 gene [22]. However, PARP cleavage was observed in both cell lines suggesting that it could have proceeded via activation of another effector caspase in MCF-7 cells. These evidences conclude that the mitochondria-mediated pathway is a possible route of apoptosis in HL-60 and MCF-7 cells upon treatment with fucoidan purified from *S. polycystum*. While caspases are considered key biomarkers of apoptosis, numerous other apoptogenic molecular mediators, which do not require caspase activation, have been discovered as potential targets for anticancer drugs. Mitogen-activated protein kinases are a major grouping of such proteins involved in mediating apoptosis, which could be targeted by fucoidans [23]. Hence further analysis of F5 could widen its potential use in anticancer drug research. Structure-activity relationships of fucoidan are still a controversy where fucoidan is highly heterogeneous in its structure. A large number of studies on this topic suggest that sulfate content and molecular weight of polymer are major factors related to the antioxidant, anticancer, and immunomodulatory properties of fucoidan [24]. Present evidence suggests that fucoidans from *S. polycystum* could provide health benefits and could be utilized as candidates in cancer chemotherapy.

## 4. Materials and Methods 

### 4.1. Materials

Celluclast was purchased from Novozyme (Nordisk, Bagsvaerd, Denmark). Dulbecco’s Modified Eagle Medium (DMEM), fetal bovine serum (FBS), and penicillin-streptomycin were purchased from Life Technologies Corporation (Grand Island, NY, USA). Fucoidan standard, dextran sulfate, and chondroitin 6-sulfate molecular weight standards and 3-(4,5-dimethylthiazol-2-yl)-2,5-diphenyltetrazolium bromide (MTT), acridine orange, 2′ 7′-dichlorodihydrofluorescein diacetate (DCFH2-DA), propidium iodide (PI), trifluoroacetic acid, ethidium bromide, o-Toluidine blue, Hoechst 33342, agarose, low melting agarose, and sodium dodecyl sulfate were purchased from Sigma-Aldrich (St Louis, MO, USA). Polyvinylidin-fluorid (PVDF) membranes were obtained from Millipore (Schwallbach, Germany). All organic solvents used in this study were of the HPLC grade and purchased from Daejung Chemicals & Metals (Gyeonggi-do, S. Korea).

### 4.2. Collection and Extraction of Fucoidan Rich Fraction

Following up on the preliminary screening [25], *Sargassum polycystum* samples for the mass extraction were collected from the south-west coast of Sri Lanka. After washing with tap water, the samples were lyophilized and ground into a powder. The algae powder (350 g) was suspended in a solution of 95% ethanol for depigmentation (repeated three times). Depigmented powder was suspended in a solution of 10% formaldehyde in ethanol for 8 h at 37 °C. After filtration, the powder was washed twice with 95% ethanol and dried at 45 °C in a drying oven and lyophilized to remove any remaining moisture and ethanol. The powder (300 g) was then suspended in sterilized distilled water, and the pH was adjusted to 4.5 using 1 M HCl. Celluclast was added to the suspension, reaching a concentration of 0.5% of the substrate concentration. The mixture was incubated with continuous agitation at 50 °C for 24 h while regulating the pH at 4.5. After the digestion, the mixture was filtered through cheesecloth, and the filtrate was centrifuged to remove any particles. Celluclast was heat deactivated by incubating the supernatant at 100 °C for 10 min. The supernatant pH was adjusted to 8.0. Alcalase (0.1%, for 8 h at 50 °C) was used to hydrolyze the proteins. Alcalase was heat-inactivated by the same method above. Then the mixture was acidified to a pH of 4.0 using HCl and gradually mixed with a solution of saturated CaCl_2,_ facilitating the precipitation of alginate as calcium alginate. The mixture was then centrifuged, obtaining the supernatant. The supernatant was neutralized by using NaOH and lyophilized to reduce the water content to 1/4^th^ of the initial extraction volume and mixed with four volumes of 95% ethanol to precipitate the polysaccharides.

### 4.3. Fucoidan Purification

The precipitated polysaccharides were purified using an anion exchange chromatography on an ÄKTA chromatographic system (Uppsala, Sweden). The column (DEAE-sepharose) was equilibrated with 50.0 mM sodium acetate buffer (pH 5.3) and eluted with gradient elution using a solvent of 50.0 mM sodium acetate buffer with increasing amounts of 2.0 M NaCl in the same buffer. The eluates were collected into a hundred 15 mL conical tubes. The polysaccharide content in each tube was measured by the phenol-sulfuric assay [26].

### 4.4. Chemical Analysis

The proximate composition of the chemical constituents in the samples was determined according to the methods described in AOAC 2002 [27]. The adopted AOAC methods for the evaluation of ash, protein, lipid, and carbohydrates were dry ashing in a furnace at 600 °C for 5 h, Kjeldahl digestion, Soxhlet extraction, and phenol-sulfuric assay. The polysaccharide and sulfate content of the purified fractions were respectively analyzed using the standard phenol-sulfuric assay and BaSO_4_ precipitation methods with some minor modifications [28]. Additionally, the protein and polyphenol content were estimated using the commercial BCA protein assay kit and by Folin–Ciocalteu method, respectively [29]. Monosaccharide composition was analyzed by a CarboPac PA1 cartridge column connected to an ED50Dionex electrochemical detector after hydrolysis by trifluoroacetic acid [1].

### 4.5. FTIR and NMR Analysis

FTIR is extensively used for the structural characterization of polymers as it allows identification of the functional groups. 10 mg of the purified polysaccharide powder were mixed with KBr and cast into pellets. The FTIR readings were taken using a Thermo Scientific NicoletTM 6700 FTIR spectrometer, MA USA. For the NMR analysis, the dialyzed polysaccharide was lyophilized, and deuterium exchanged with D_2_O. The samples were then dissolved in D_2_O, and a minute amount of deuterated dimethyl sulfoxide was added as an internal standard. NMR spectra were obtained using a JEOL JNMECX400, NMR spectrometer, Japan at 33 k.

### 4.6. Evaluation of Molecular Weight Distribution

The molecular weights of the purified fractions were analyzed using agarose gel electrophoresis following our previous method [1]. Briefly, the polysaccharide samples were electrophoresed in 1% agarose gels using a running buffer composed of Tris-Borate-EDTA (pH 8.3). The electrophoresis was carried out for 20 min at 100 V. The gel was stained with 0.02% o-Toluidine in 3% acetic acid with 0.5% Triton X-100 and destained with 3% acetic acid. Molecular weight markers used were, dextran sulfate (MW 50–500 kDa), chondroitin 6-sulfate (MW 60 kDa), Dextran sulfate (MW ≈ 50 kDa) and Dextran sulfate (MW ≈ 8 kDa).

### 4.7. Cell Culture

The MCF-7 cells were cultured in DMEM media and HL-60, and Vero cells were cultured in RPMI media, both of which were supplemented with 10% FBS and 1% penicillin/streptomycin mixture. Cell cultures were maintained under 37 °C in a humidified atmosphere supplemented with 5% CO_2_. The cells under exponential growth were seeded for the experiments. The cancer cells were seeded in a concentration of 1 × 10^5^ cells mL^−1^ in 96 well culture plates for 24 h and treated with different concentrations of the samples. After incubating for 24 h, the MTT assay was carried out for the determination of cell viability [30].

### 4.8. Evaluation of Nuclear Morphology

Nuclear fragmentation and chromatin condensation of the cancer cells were evaluated by using the nuclear staining dye Hoechst 33342 (10 μg mL^−1^) and via the double staining method using acridine orange/ethidium bromide (100 μg mL^−1^). Experiments were carried out, according to Fernando et al. (2018) [21]. Briefly, 24 h pre-seeded cells were treated with different concentrations of the samples and incubated for 24 h. The fluorescence dyes were then applied to the wells and incubated for 10 min. The images were visualized using a fluorescence microscope with a CoolSNAP-Pro color digital camera.

### 4.9. Cell Cycle Analysis

The proportion of cells in the Sub-G_1_ phase of the cell cycle, which indicate apoptotic hypodiploid cells were measured using flow cytometry. The experiments were carried out according to the method described by Fernando et al. (2018) [21]. Briefly, cells (2 × 10^5^ cells mL^−1^) were incubated with different concentrations of F5 for 24 h. The cells were then harvested, washed with PBS, and fixed in 70% ethanol followed by washing with EDTA (2 mM) in PBS. Then the cells were suspended in a PI solution containing RNase, and the cell cycle analysis was carried out using a FACSCalibur flow cytometer, USA.

### 4.10. Analysis of DNA Damage

The DNA damage caused by F5 was analyzed by evaluating the single-cell DNA damage (comet assay) and DNA laddering analysis. The alkaline comet assay was performed by using cells incubated with different concentrations of F5 for 24 h. Experiments were carried out according to the method described by Fernando et al. (2018) [21]. For the DNA laddering analysis, the cells were incubated with samples for 24 h and harvested after PBS washing. The experiments were performed following the method described by Jayasooriya et al. (2012) [31].

### 4.11. Western Blot Analysis

The cells were pre-seeded in 6 well culture plates at 2 × 10^5^ cells mL^−1^ for 24 h and treated with different sample concentrations. After 24 h, the cells were harvested, lysed, and centrifuged at 12,000× *g* for 20 min to remove the cellular debris. The proteins were resolved by SDS-polyacrylamide jell electrophoresis. Protein bands were blotted onto nitrocellulose membranes and incubated for 2 h with skim milk in TBST. The membranes were consequently incubated with primary (8 h under 4 °C) and secondary antibodies (2 h at room temperature). Protein bands were visualized by adding chemiluminescent substrate (Cyanagen Srl, Italy) using a FUSION SOLO Vilber Lourmat system, France [21].

### 4.12. Statistical Analysis

All data values are expressed as means ± SD based on at least three independent determinations. Significant differences among the data values were determined using IBM SPSS Statistics 20 software using one-way ANOVA by Duncan’s multiple range test. ** P-values less than 0.05 (*p* < 0.05) were considered significant.

## Figures and Tables

**Table 1:** The composition of column fractions.

		F1	F2	F3	F4	F5
Yield (%)		32.52 ± 0.17	12.24 ± 0.14	9.36 ± 0.09	11.78 ± 0.07	34.09 ± 0.14
Chemical composition (%)	Polysaccharide	81.42 ± 0.09	75.66 ± 0.11	70.25 ± 0.26	64.82 ± 0.24	59.36 ± 0.03
	Sulfate	12.42 ± 0.30	18.75 ± 0.00	23.12 ± 0.29	28.61 ± 0.22	33.56 ± 0.07
	Protein	0.36 ± 0.02	0.28 ± 0.01	0.25 ± 0.02	0.33 ± 0.03	0.28 ± 0.00
	Polyphenol	0.52 ± 0.02	0.55 ± 0.00	0.48 ± 0.04	0.39 ± 0.01	0.27 ± 0.02
Monosaccharide composition (%)	Fucose	20.06	33.20	49.06	63.84	71.96
	Rhamnose	1.72	1.45	0.89	0.60	N.D.
	Arabinose	2.52	2.39	1.15	0.62	N.D.
	Galactose	33.32	27.64	22.64	17.39	12.31
	Glucose	7.48	5.81	3.74	1.46	1.41
	Mannose	32.67	26.29	N.D.	N.D.	N.D.
	Others	2.22	3.22	22.52	16.09	14.32

Chemical composition was calculated based on triplicate determinations. Results are given as the means ± SD.
